# The Hahnemann Monument

**Published:** 1899-01

**Authors:** Bushrod W. James

**Affiliations:** N. E. Cor. 18th and Green Streets; Philadelphia


### THE HAHNEMANN MONUMENT.

N. E. Cor. 18th and Green Streets.

PHILADELPHIA, November 29th, 1898.

EDITOR OF THE HOMŒOPATHIC PHYSICIAN:

I enclose you a list of the paid American subscriptions to the International Monument Fund, for the restoration of Hahnemann's tomb, as credited in the *Revue Homœopathique Francaise*, Paris, France.

Dr. F. Cartier, the Secretary of the Commission, only acknowledges, in this way, the subscriptions when accompanied by the cash. The whole amount in hand thus far (October) is 10,654.35 francs, and it is the desire of the French Society to have 15,000 francs in hand before contracting for the work.

If you have space I should be glad to have you publish this list in an early number of your journal.

I am very truly and fraternally yours,

BUSHROD W. JAMES.

American subscribers to the International Monument Fund, for the restoration of Hahnemann's tomb.

Subscriptions received through Dr. Bushrod W. James, and amounts sent to Dr. F. Cartier, Paris.

| | | |
|---|---|---|
| Dr. W. P. Wesselhoeft, | $100 00 | |
| Massachusetts Surgical and Gynæcological Society, | 50 00 | |
| Dr. John McE. Wetmore, | 25 00 | |
| Dr. Bushrod W. James, | 20 00 | |
| Alumnæ Association of the N. Y. Medical College and Hospital for Women, | 12 00 | |
| Dunham Medical Club of Hartford, Conn., | 6 00 | |
| Dr. Henry E. Spalding, | 5 00 | |
| Dr. L. Dennis, | 5 00 | |
| Dr. Walter Wesselhœft, | 5 00 | |
| Dr. Thomas Shearer, | 5 00 | |
| Dr. Thomas L. Shearer, | 5 00 | |
| Drs. C. H. & C. D. Martin, | 5 00 | |
| Dr. W. L. M. Fiske, | 5 00 | |
| Summit Co. (Ohio) Clinical Society, | 5 00 | |
| Dr. Jas. Harwood Closson, | 3 00 | |
| Dr. H. C. Allen, | 2 00 | |
| Dr. E. H. Orme, | 1 00 | |
| Dr. J. W. Hayward, | 1 00 | |
| Miss I. Florence Himmelwright, | 1 00 | |
| | $261 00 | |
| | | Francs. |
| | | 1,316 60 |
| Dr. Tysdale Talbot (paid direct to Dr. Cartier), | | 20 00 |
| Dr. A. B. Norton (paid direct to Dr. Cartier), | | 20 00 |

